# Supplementary figures and images for: Vesicle-mediated transport of ALIX and ESCRT-III to the intercellular bridge during cytokinesis
Source: Cell Mol Life Sci. 2023 Jul 31;80(8):235. doi: 10.1007/s00018-023-04864-y (PMC10390626; doi:10.1007/s00018-023-04864-y)

Suppl. Figure 1

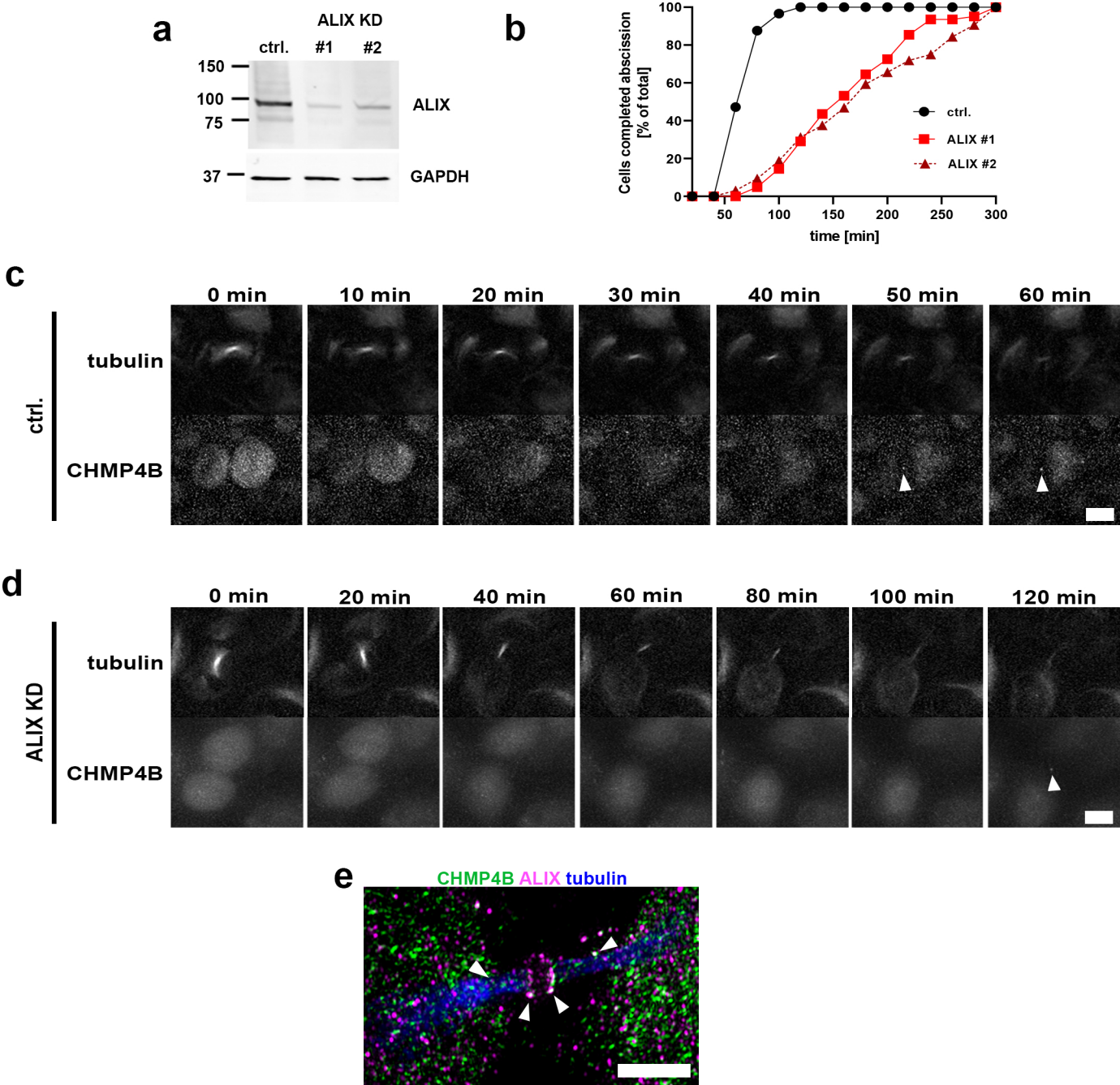

Suppl. Figure 2

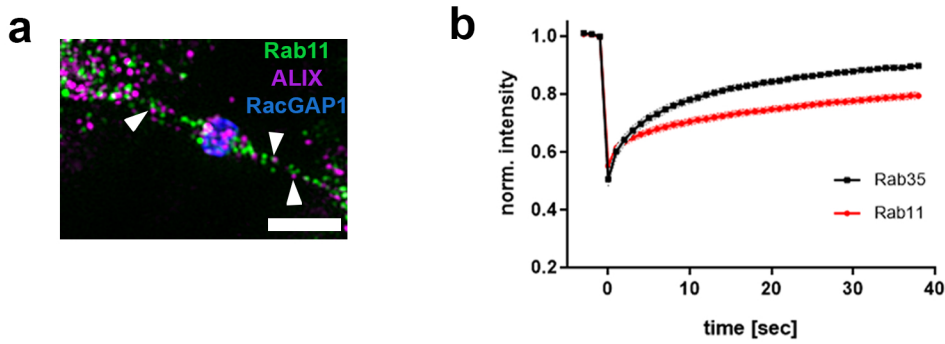

Suppl. Figure 3

a

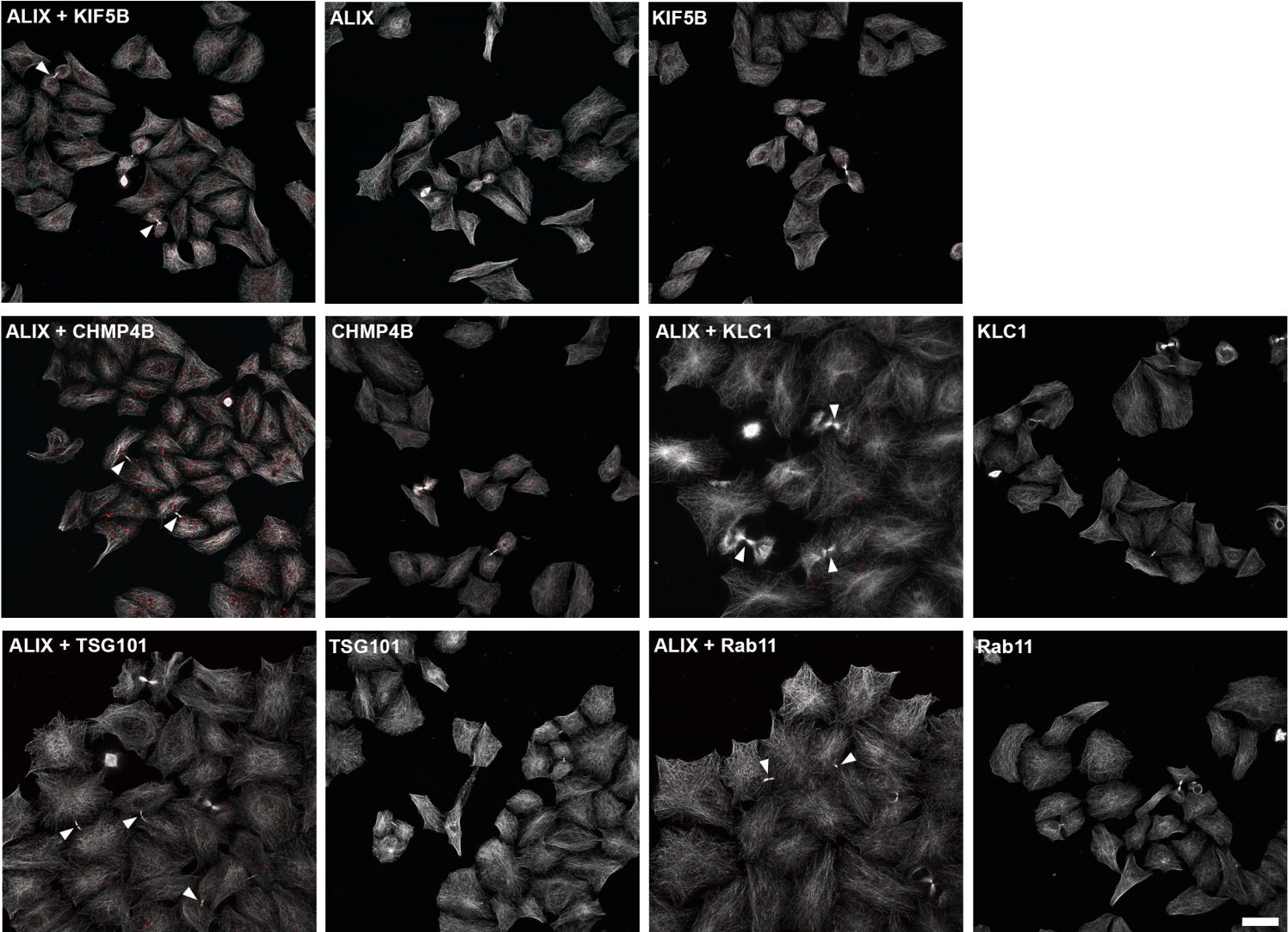

b

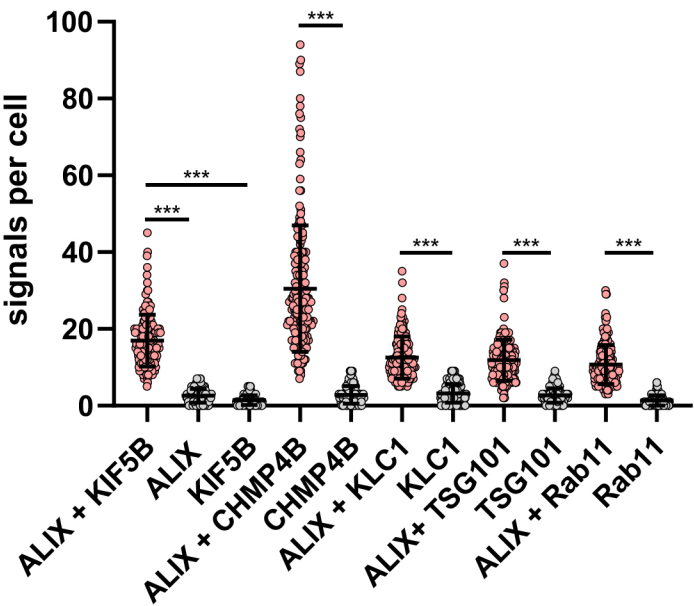

# Suppl. Figure 4

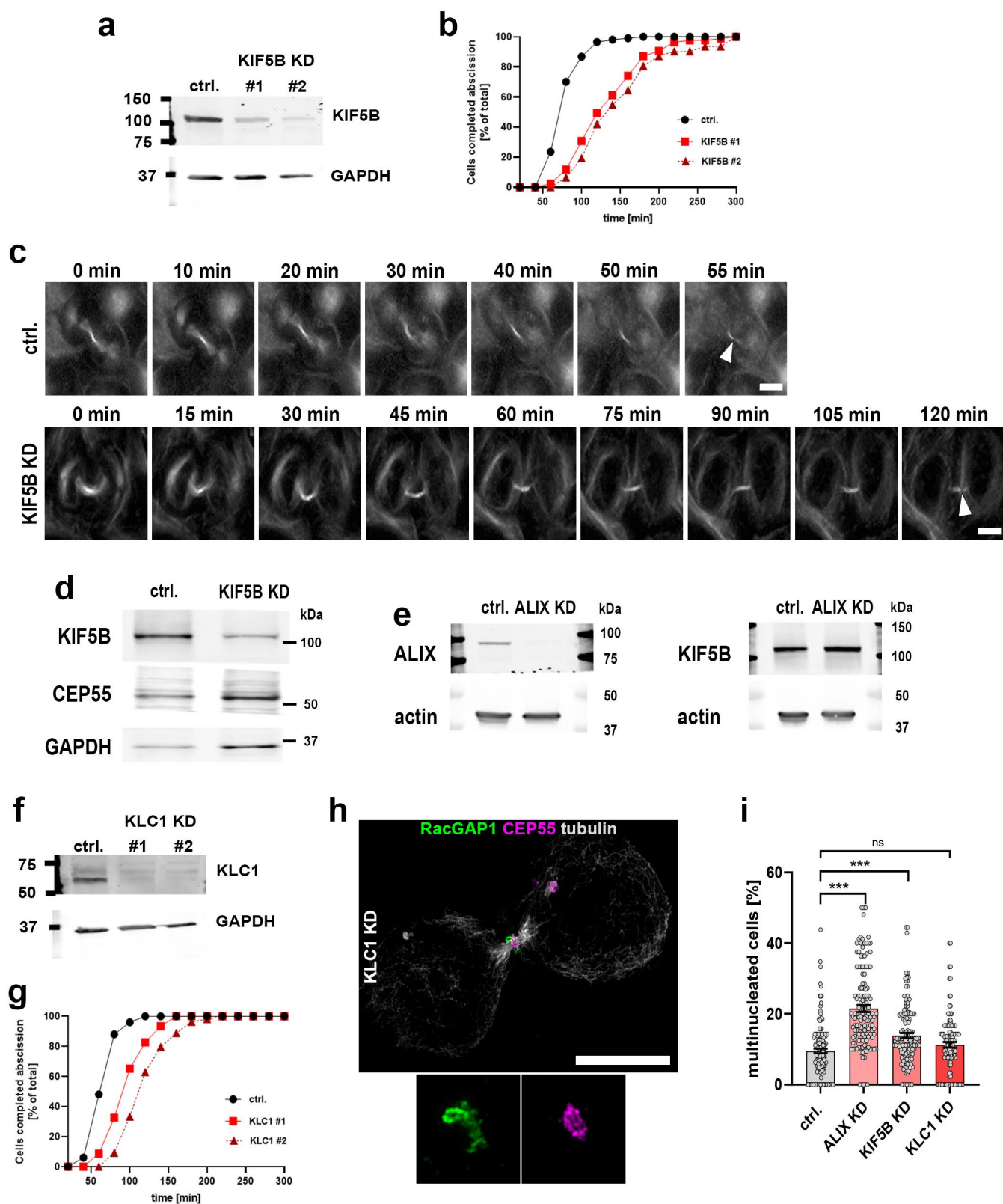

Supplement: Supplementary file 1 — Supplementary file1 Supplementary Fig. 1. Delayed abscission and recruitment of CHMP4B to the midbody upon ALIX knockdown. (a) Western blot showing knockdown (KD) efficiency of siRNA-induced ALIX depletion after 3 days of transfection with two different siRNA oligos (ALIX #1 oligo = ALIX oligo shown in Fig. 2a). (b) Cumulative frequency plot showing the time interval between ICB formation and abscission upon control and ALIX siRNA treatment (with two different oligos, #1 and #2) as indicated (ctrl.: n =130; ALIX KD #1 n=115; ALIX KD #2: n=94 cells from three independent experiments; control: 65.96 ± 1.41 min; ALIX KD #1: 156.7 ± 5.73 min; ALIX KD #2: 148.4 ± 6 min [mean time 50% of cells completed abscission ±SEM]; P < 0.001). (c -d) Selected frames from time-lapse microscopy movies of cells stably expressing CHMP4B-GFP and labelled with SiR-tubulin at indicated time points. (c) Recruitment of CHMP4B to the midbody during cytokinesis in control (ctrl.) cells. The upper panel shows SiR-tubulin, visualizing an ICB, and the bottom panel visualizes the CHMP4B signals. Arrowheads indicate the first appearance of CHMP4B-GFP at the midbody starting at 50 min after the formation of a stable ICB. (d) Recruitment of CHMP4B to the midbody during cytokinesis in ALIX KD cells. The upper panel shows SiR-tubulin and the bottom panel visualizes the CHMP4B signals. The arrowhead indicates the first appearance of CHMP4B-GFP at the midbody starting at 120 min after the formation of a stable ICB. Scale bars = 10 µm. (e) Close proximity of endogenous ALIX and CHMP4B in the cytokinetic bridge. SIM micrographs of fixed cells stained for ALIX (magenta), CHMP4B (green) and tubulin (blue) showing ALIX and CHMP4B proximity in vesicular structures in the ICB and at the midbody (indicated by arrowheads). Scale bar = 3 µm. Supplementary Fig. 2. ALIX and Rab11 in the cytokinetic bridge and FRAP analysis of Rab35 and Rab11 dynamics at the midbody during cytokinesis. (a) Close proximity of endogeno [file 18_2023_4864_MOESM1_ESM.pdf]
